# Birth Weight, Cord Blood Lipoprotein and Apolipoprotein Levels in Indian Newborns

**Published:** 2010

**Authors:** Simmi Kharb, Ramanjit Kaur, Veena Singh, Krishna Sangwan

**Affiliations:** 1 Department of Biochemistry, Pt BDS PGIMS, Rohtak, India; 2 Department of Obstetrics and Gynecology, Pt BDS PGIMS, Rohtak, India

**Keywords:** Cord blood, Fetal period, Lipoprotein, Apolipoprotein, Primordial prevention

## Abstract

**Objectives:**

Primordial prevention of chronic disease is of clinical and public health importance. Considering the fetal onset of atherosclerosis, we aimed to determine the cord blood level of lipoproteins and apolipoproteins as well as their correlation with birth weight and gestational age.

**Methods:**

This cross-sectional study comprised 100 healthy Indian newborns. Ten ml. of cord blood was collected from placental end of umbilical vein. Serum was separated by centrifugation and analyzed on the same day for lipid profile including total cholesterol (TC), triglycerides (TG), high density lipoprotein- cholesterol (HDL-C), very low density lipoproteincholesterol (VLDL) and low density lipoprotein-cholesterol (LDL-C), apolipoproteins A-I and B (ApoA-I, ApoB). Atherogenic index (AI) was calculated as the ratio of ApoB to ApoA-I.

**Results:**

Cord blood of female newborns had higher TC, HDL-C, LDL-C, Apo A-I, Apo B and AI as compared to male newborns, whereas TG and VLDL-C were higher in male than in female newborns. Significant positive correlation was observed between cord blood Apo A-I and HDL-C (r= 0.45, p<0.01), and between cord blood Apo-B and LDL-C (r= 0.44, p<0.01). Non-significant inverse correlation was observed between Apo A-I and ApoB with gestational age. There was a significant inverse correlation between TG and gestational age (r= –0.197, p <0.05). Positive non-significant correlation was observed between AI and birth weight (r=0.046, p>0.05).

**Conclusions:**

These findings are another confirmatory evidence for the association of prenatal factors with cord blood lipid profile, and can serve as starting point for studying lipid transport system changes during early life.

## INTRODUCTION

Atherosclerosis, a major cause of cardiovascular disease, is a process that begins early in life and progresses silently for decades.^1^ Epidemiological studies reveal a strong independent relationship between serum cholesterol and coronary heart disease.^2^ Maternal hypocholesterolemia has been reported to enhance early atherogenesis and marked increase in fatty streaks in human fetuses.^3^

A fetus needs a considerable amount of cholesterol for development of tissues and organs. After birth, human lipid transport system is transformed from one containing low levels of very low density lipoprotein (VLDL) and low density lipoprotein-cholesterol (LDL-C) to adult pattern with relatively high levels of LDL-C which continues to increase with age. Cord sera have been demonstrated to contain all well-characterized adult lipoproteins and apolipoproteins.^4^ Fetal growth restriction is associated with a chronic pattern of atherogenic lipoprotein metabolism. The relative amount of apolipoproteins A-I and B (ApoA-I, ApoB) is important. Atherogenic index (AI), i.e. the ratio of ApoB to ApoA-I, is found to track closely during the first year of life.^5^ Abnormal lipoprotein profiles in childhood persist into adult life and elevated Apo B levels in young adults have been linked to atherosclerosis in later life.^5^ Young age can be viewed as an opportunity to begin preventive interventions to change risk factors for cardio-vascular risk. Hence the present study was planned to analyze cord blood lipoproteins and apolipoproteins levels and to correlate them with birth weight and gestational age.

## METHODS

The present study was conducted in the Department of Biochemistry in collaboration with Department of Obstetrics and Gynecology, Pt.B.D.Sharma PGIMS, Rohtak, India. The study protocol was approved by the institute’s Committee on Human Research and the experiments conformed to institutional standards. Mothers gave their informed consent. The study group consisted of 100 healthy neonates born following healthy normotensive pregnancy and normal termdelivery.

Ten milliliter of cord blood was collected from the placental end of umbilical vein. Serum was separated by centrifugation and analyzed on the same day for lipid profile, including total cholesterol (TC), triglycerides (TG), HDL cholesterol (HDL-C), VLDL and LDL-C, as well as ApoA-I and ApoB.^6^–^11^ AI was calculated as the ratio of ApoB to ApoA-I.^12^

*Statistical analysis:* Continuous data was computed as mean ± standard deviation (SD). The Student’s t-test was applied for comparison of mean values. The relationship of cord blood lipid profile with birth weight and gestational age was determined by regression analysis. Data were analyzed using the SPSS statistical package version 14.0 for windows (SPSS Inc., Chicago, USA). The significance level was set at p<0.05.

## RESULTS

In female newborns, TC, LDL-C (p<0.01, in both parameters) and HDL-C (p<0.05) were significantly higher in comparison to their male counterparts. TG and VLDL-C levels were higher in male newborns as compared to females but the difference was not statistically significant (Table 1).

**Table 1:** Mean (SD) level of lipid profile in cord blood (mg/dL)

	Total	Male	Female
	n=100	n=50	n=50
Total Cholesterol (mg/dL)	73.64+21.61	68.38+16.69	78.90+24.68^*^
HDL-C (mg/dL)	23.25+7.66	21.66+6.78	24.84+8.22^**^
LDL-C (mg/dL)	41.81+17.88	37.76+6.78	45.86+18.95^**^
VLDL-C (mg/dL)	12.81+11.08	13.35+12.45	12.16+9.47^+^
Triglycerides (mg/dL)	33.75+16.39	34.32+17.58	33.18+15.28^+^
Apolipoprotein A-I (mg/dL)	67.08+17.28	63.52+15.30	70.64+18.52^+^
Apolipoprotein B (mg/dL)	32.15+16.10	28.54+13.78	35.76+17.52^*^
Atherogenic index	0.47+ 0.16	0.45+0.15	0.49+0.16**

Significant positive correlation was observed between cord blood Apo A-I and HDL-C (r= 0.45, p<0.01), as well as between Apo-B and LDL-C (r= 0.44, p<0.01).

Table 2 presents the relationship of cord blood lipid profile with birth weight and gestational age. Apo A-I and Apo-B had a non-significant inverse correlation with gestational age. TG had significant inverse relationship with gestational age(r= –0.197, p <0.05). A non--significant positive association was observed between AI and birth weight (r=0.046).

**Table 2:** Relationship of apolipoproteins and triglycerides with gestational age

	r	p value
Apo A-I	-0.056	NS
Apo-B	-0.039	NS
Triglycerides	-0.197	<0.05

NS: Not significant

## DISCUSSION

In this study, cord blood levels of TC, HDL-C and LDL-C were significantly higher in females than in males. Although cord blood levels of TG and VLDL- C were higher in females than in males, but this difference was not statistically significant. Our findings are in agreement with some previous studies^13^,^14^ and suggest that gender-related factors might influence lipid levels from fetal period.

It is well-documented that low Apo A- I and / or increased Apo B are associated with increased cardiovascular risk. Elevated LDL- C and Apo B levels in young adults are linked with cardiovascular disease in later life.^12^ In the present study, Apo A-I, ApoB and AI were higher in female cord blood as compared to males. In cord blood, all well-characterized adult apolipoproteins with reduced ApoB have been reported.^4^ Moreover, we found significant positive correlation between Apo A-I and HDL-C, as well as between ApoB and LDL-C. Our findings are in agreement with those reported by Sattar et al.^15^ Gender-based differences in lipoprotein metabolism have been reported to be implicated in lipoprotein metabolism.^13^,^14^,^16^

A link between low birth weight and adult onset atherosclerosis has been reported in literature, where elevated Apo B levels were observed in growth-retarded fetuses.^17^ Levels of Apo A-I and ApoB have been reported to be measured in fetuses with normal growth, data are available regarding circulating fetal levels of apolipoproteins in growth retarded fetuses. These findings indicate that apo-B levels are elevated in growth-retarded fetuses and might be considered as confirmatory evidence on a link between low birth weight and adult onset atherosclerosis. Radunovicetal et al. reported significant differences in ApoB level and its ratio to Apo A-1, i.e. AI in growth-retarded fetuses as compared to normal fetus. They also reported that fetal Apo A-I and ApoB levels do not correlate with gestational age.^18^ In the present study, Apo A-I and ApoB levels had non-significant inverse association with gestational age, whereas TG had a significant negative correlation with gestational age. Fetal growth retardation establishes a life-long irreversible atherogenic profile and those individuals with a history of low birth weight are reported to have an atherogenic profile.^18^ In the present study, AI showed positive correlation with birth weight. Barker et al. have reported an inverse correlation of birth weight and neonatal abdominal circumference with adult serum cholesterol, LDL-C and Apo B levels, suggesting that the association between aberrant lipoprotein metabolism and low birth weight is present by the time intrauterine growth restriction is clinically evident.^19^ Other reports have demonstrated that abnormal lipoprotein profiles in childhood persist into adult life.^20^,^21^ Furthermore, the prevalence and severity of carotid artery atherosclerosis in later life are linked to lower birth weights.^22^ A Swedish cohort study found a strong relationship between impaired fetal growth and subsequent cardiovascular mortality.^23^ These findings indicate that fetal growth restriction is associated with a chronic pattern of atherogenic lipoprotein metabolism. Lack of any significant difference in total TG levels between the two groups in the present study supports a constitutional basis for the differences in apolipoprotein metabolism, observed in our study.

Immunochemical studies of the cord lipid transport system have shown that all the well-characterized apolipoproteins are present in cord blood, although at lower level than that found in the normal adult. McConathy et al. reported existence of all well-characterized adult apolipoproteins in cord blood, with Apo B levels most reduced.^4^ In the present study, we also observed that apo B level were most reduced of the apolipoprotein parameters in cord blood, and AI was higher in cord blood of female newborns as compared to their male counterparts.

Fetus needs a considerable amount of cholesterol for development of tissues and organs, there should be no surplus cholesterol.^24^ However, high AI is found to track closely during the first year of life.^25^ Thus, it is suggested that atherogenic milieu occurring during pregnancy persists into adulthood and fetal growth retardation is associated with adult atherosclerosis.

## CONCLUSIONS

Roots for many adult diseases, including atherosclerotic cardiovascular diseases, begin in childhood and clustered risk factors track from childhood to adulthood. These factors can be altered favorably and can be modified by preventive interventions and early lifestyle related factors. Establishment of references values for lipoprotein and apolipoprotein would facilitate comparison between different studies. Data on lipoproteins, apolipoprotein presented here can serve as starting point for studying lipid transport system changes and thyroid hemostasis during early days and months of life and for correlating them with cardiovascular disease. These findings are another confirmatory evidence for the association of prenatal factors with cord blood lipid profile, and can serve as starting point for studying lipid transport system changes during early life.
